# Design of Electro-Thermal Glove with Sensor Function for Raynaud’s Phenomenon Patients

**DOI:** 10.3390/ma14020377

**Published:** 2021-01-14

**Authors:** Hewan Dawit, Qian Zhang, Yimeng Li, Syed Rashedul Islam, Jifu Mao, Lu Wang

**Affiliations:** 1 Key Laboratory of Textile Science & Technology of Ministry of Education and College of Textiles, Donghua University, 2999 North Renmin Road, Songjiang District, Shanghai 201620, China; hewan27dawit@gmail.com (H.D.); zh901026@163.com (Q.Z.); yimeng_u@163.com (Y.L.); sri060791@gmail.com (S.R.I.); 2 Key Laboratory of Textile Industry for Biomedical Textile Materials and Technology, Donghua University, Shanghai 201620, China

**Keywords:** Raynaud’s phenomenon, electro-thermal property, strain sensor, contact resistance, knitted structure

## Abstract

Raynaud’s phenomenon (RP) is a disease that mainly affects human fingertips during cold weather. It is difficult to treat this disease using medicine, apart from keeping the body in a warm environment. In this research, conductive knitted fabrics were fabricated to help relax the vessels of the patient’s fingertips by providing proper heat, and also serving as a sensor to detect finger motion after relaxation of the blood vessels of patients. Four different structures, termed plain, purl, interlock, and rib were produced using conductive silver/PE (polyethylene) yarn and wool yarn, with a computerized flat knitting machine. The effect of knitted structure on the electro-thermal behavior, sensitivity, and stability of resistance change (∆R/R) under different tensile forces was investigated. By comprehensive comparison, the purl structure was identified as the preferred structure for the heating glove for RP patients, owing to superior electro-thermal behavior. Additionally, the purl structure had a greater capacity to detect different motions with stable resistance change. This potential electro-thermal glove could be used for functional, as well as aesthetic (fashion) purposes, and could be worn at any time and occasion with complete comfort.

## 1. Introduction

Raynaud’s phenomenon (RP) is a disorder resulting in vasospasm which occurs because of the contraction of a blood vessel. It is a particular series of discolorations of the fingers and/or the toes because of limited transportation (flow) of blood to the fingers after exposure to change in temperature or emotional events [1,2,3,4]. In cold temperatures, the body automatically takes the warm blood from the extremities and draws it toward the core, where it can keep the heart, lungs, and other organs protected [5]. However, in RP patients the flow of blood to the fingertips is restricted due to narrow blood vessels of the fingertips [2,6,7]. Even though several studies have been made to cure the disease, there is no approved cure for Raynaud’s disease, but there are ways to manage symptoms. The main recommended method to manage this disease is keeping the hand warm using hot water to relax the vessels. However, it is an inconvenient method to use hot water always and everywhere. Thus, using a glove is much more convenient and can reduce the pre-pain felt by the patients. Though heating gloves have been developed by several researchers, the gloves were not explicitly for RP patients, and are not comfortable to be worn at any time and place. Some of the recently reported heating gloves, such as in Seung-Won. K et al. [8] and Colin V et al. [9], were manufactured specifically for virtual reality applications. Although in the work of Seung-won the glove had electrothermal behavior, the glove design was not precisely for a heating glove, as it could not cover the whole finger and the materials used could affect the comfort of the wearer.

Knitting fabrics are the most common textiles used in applications to provide warmth, flexibility, and comfort. Knitting fabric is formed by intermeshing loops of a single yarn, or set of yarns, together [10].

It has become a promising structure in textile technology because of the flexibility, comfort, and warmth that the structure provides. For that reason, it is a good candidate for a variety of uses in medical applications and other areas that integrate electronic devices [11,12]. Though a knitted fabric can provide warmth and flexibility, it is not enough to adapt special properties such as electro-thermal and sensing behaviors to wearable smart textiles if produced separately. The fabric must be aimed at the required, technical, and exceptional applications, such as the amount of heat needed by RP patients, which is different from the heat a normal person requires. The amount of heat for RP patients should be able to relax the blood vessels of the patient’s finger. Meanwhile, there has been increasing research on heating electro-thermal knitted conductive fabrics that can convert electrical energy into heat without affecting the human wearing experience. Therefore, knitted fabric electrical heaters have the potential to be used as medical heat therapy, joint pain relief, and athletic rehabilitation [13,14].

Electrical conductivity in the knitted fabric can be achieved in many ways, depending on the desired level of conductivity, durability, and the application. One way to enhance the conductive properties of a knitted fabric is to blend it with conductive materials or polymers. Several ways have been presented on how to intermix a conductive material with a non-conductive material. The common method to assimilate knitted fabric with conductive materials is by coating the conductive components onto the existing fabrics [15]. Dandan. H et al. [16] introduced a conductive fabric by coating knitted fabric using Polypyrrole to construct wearable textile sensors. Rujun. M et al. [17] also manufactured conductive knitted fabrics with poly(dimethylsiloxane) coated fibers for application in strain sensing. Similarly, Hyelim. K et al. [18] reported an electro-conductive para-aramid knit, manufactured by dip-coating in a graphene/waterborne polyurethane (WPU) composite for a use as a heating fabric and protective clothing. 

It is a simple and convenient method to fabricate a coated fabric using conductive polymers. However, coated fabrics usually perform poorly due to the impairment of the conductive circuits during large mechanical deformations [19]. In addition to the heating textiles, conductive textiles as sensors are also the most explored material in biomedical applications, for detecting different parts of the body. Strain sensors play significant roles in biomedical electronics, and can monitor various body signals, including physical, chemical, and biological signals [20]. 

Therefore, in this work, an electro-thermal with a sensing function was amalgamated to give a multifunctional advantage to RP patients. The glove was designed with sensing functionality to help the patients detect the movement after relaxing the fingertips. It could be used as a functional and fashionable glove, combined. A conductive fabric was investigated to optimize a suitable knitted structure that could be used as a multifunctional heating glove, and sensing the movement of the patient’s finger. The wool yarn was integrated with silver/PE (polyethylene) conductive yarn to enhance the electro-thermal behavior of the fabric. Four different weft knitted structures named plain, purl, interlock, and rib were manufactured to study the effect of the structure on the resistance change (∆R/R) and electro-thermal behavior of each structure. A suitable knitted structure that can be used as a heating textile and sensor was identified after a series of experiments. Based on the optimization, an electro-thermal glove with a sensor function was designed for RP patients.

## 2. Material and Methods

### 2.1. Materials

In this experiment, silver/PE (polyethylene) yarn and 100% pure wool yarn were utilized to produce four different conductive knitted fabrics. The silver/PE yarn was purchased from a market supplier (Suzhou TEX Silver fiber technology CO, Ltd., Suzhou, China) and the wool yarn was purchased from Hebei ASEAN Cashmere products Co, Ltd. (Hebei, China).

Due to its good thermal insulation, wool yarn was used as a material for thermal insulating purposes and to give comfort to the patient. Silver/PE yarn was used as a conductive yarn and to produce heat through the given power. The tensile strength of both yarns was examined using a universal mechanical tester, YG028 (Ningbo Dahe instrument Co., Ltd., Ningbo, China). A gauge length of 50 mm with a speed of 10 mm/min was used for both yarns. The morphology of silver/PE and wool was observed under scanning electron microscopy (SEM, Hitachi S-4800, Tokyo, Japan) as presented in Figure S1. Table S1 shows the parameters of the yarns.

### 2.2. Preparation of Knitted Fabric

**Figure 1:**
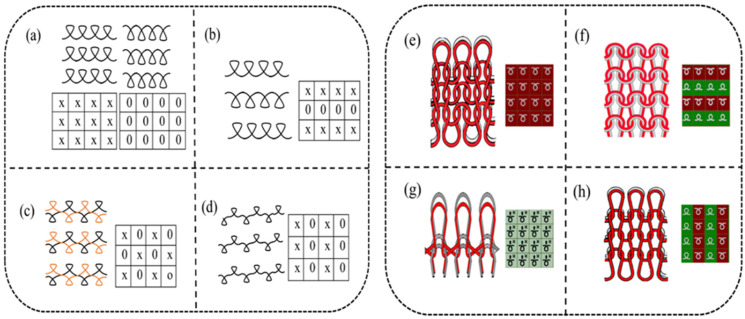
Structural notation, (**a**–**d**) plain, purl, interlock, and rib, respectively, (**e**–**h**) loop diagram and simulation effect.

Four kinds of knitted structure samples were manufactured using conductive and non-conductive yarn. The yarns were fed to the machine at the same time parallelly. The structures were produced using a computerized flat knitting machine (Longxing, Jiangsu Jinlong technology Co, Ltd., Jiangsu, China), with a needle gauge of 14 G. Based on the structure’s design and geometry, three structures were produced with a double set of needles (purl, interlock, and rib), and one structure was produced with a single set of needles (plain), which is the most common and basic structure. The structural notation, loop diagram, and simulation effects of each sample are presented in Figure 1. The fabric morphology was observed using Vistar-Image (Nikon Corporation, Shanghai, China).

### 2.3. Characterization

#### 2.3.1. Change in Resistance and the Effect of Knitted Loop Contact

The ∆R/R of the samples under different tensile forces were carried out by using a universal tensile testing machine (Wenzhou darong textile Instrument Co. Ltd, Wenzhou, China) and system source meter (Keithley 2401B, Tektronix Company, Cleveland, Ohio), which were connected with a computer. During the elongation processes, the clamping pressure was 50 N and the two sides of the samples were covered by copper electrode blocks to observe precise results in ∆R/R. The applied tensile force was produced at a constant speed of 50 mm/min. The ∆R/R of each structure was measured from a minimal tensile force of 1%, up to 5%, 10%, 15%, and 20%.

#### 2.3.2. Electro-Thermal and Temperature Distribution of the Knitted Structure

To perceive the electro-thermal performance and temperature distribution of the structures, an adjustable DC power supply (Wuxi Anais Electronic Technology Co. LTD Wuxi, China) was used under different voltages to produce power (heat). The temperature distribution of each sample was recorded using a FLIR infrared camera at a voltage value from 1 V to 3 V (1 V, 1.5 V, 2 V, 2.5 V, and 3 V). Each sample was placed on an insulating icebox, and the infrared camera was positioned 10 cm above the samples. The temperature change was recorded to perceive the distribution and change in temperature of the samples with time and voltage. Furthermore, the temperature distribution was also observed after several cycles of washing the samples in an ultrasonic washing machine using detergent (Ethyl alcohol 99%, EtOH) and water. After washing several cycles under 40 °C of warm water, the sample was dryad in a drying machine at 37 °C, and then the temperature distribution of each sample was recorded.

#### 2.3.3. Effect of Voltage and Strain Force on the Property of the Fabric

The effect of voltage on the resistance was tested using a DC power source (Wuxi Anais Electronic Technology Co. LTD Wuxi, China) under voltage values of (1 V, 2 V, and 3 V) and the change was examined using a source meter (Keithley 2401B, Tektronix Company, Cleveland, Ohio). Moreover, the effect of strain on the electrothermal behavior of the sample was examined. Taking a sample size of 4 cm × 4 cm different tensile forces were applied using a universal tensile testing machine (Wenzhou darong textile Instrument Co. Ltd., Wenzhou, China), and the temperature distribution under different strain forces from 0% to 20% (0%, 5%, 10%, 15%, and 20%) was recorded using a FLIR infrared camera (FLIR SYSTEM AB, Shanghai, China).

## 3. Results and Discussion

### 3.1. The Structure of Conductive Knitted Fabrics

**Figure 2:**
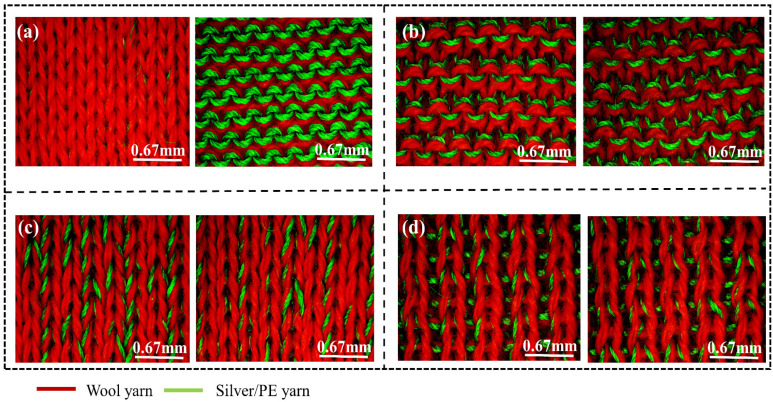
Fabric image, (**a**) Plain, (**b**) Purl, (**c**) Interlock, and (**d**) Rib.

The sample knitted fabrics were made using two yarns (wool and Silver/PE) which showed good tensile strength, as shown in Figure S2. The wool yarn could resist 8.5 MPa of stress until 20% of elongation before slippage. While the twisted Silver/PE exhibited about 195 MPa of stress until 40% of strain. Using the flat knitting machine, four weft knitted structures were produced (Figure S3). Figure 2 shows all the structures, presenting different loop formations and yarn distributions. Each structure had a different morphology. Each sample had different thicknesses, sizes, and several loops in a specific form (Table S2). During the production of the samples, the needle creates varied movements and arrangements, depending on the structure, and this led to the differentiation in the fabric morphology and yarn distribution. Meanwhile, two different yarns were used at the same time and the same content, but the appearance of both yarns on the surface of the fabric varies in each structure. Figure 2a shows that the plain structure differs on the front and back of the fabric.

The front surface of the plain structure displayed less conductive yarn compared to the backside of the fabric. The conductive yarns in the plain structure appeared on the backside of the fabric. However, the purl structure had an even distribution of both the conductive and non-conductive yarns on the front and backside of the fabric (Figure 2b). Additionally, the interlock and rib structures tended to create a high overlapping of both yarns (Figure 2c,d). The interlock structure has a behavior tending to create a sandwich structure of two fabrics. For this reason, the majority of the conductive yarns were hidden inside. However, for the rib fabric, it exhibited overlapping of yarns.

### 3.2. Effect of Knitted Structures on Resistance Change (∆R/R)

In conductive textiles, resistance is one of the most important factors, which influences the properties such as strain sensing and the thermal properties of the materials. Total resistance of conductive knitted textiles is highly dependent on two types of resistance, called length related resistance and contact resistance [21,22,23,24]. Length-related resistance is mainly decided by the length of the conductive yarn used in the fabric and the electrical conductivity of the yarn, whereas contact resistance is directly linked to the contact area and the contact pressure of the overlapped yarns [25,26,27]. It has been demonstrated that the electrical contact resistance between overlapped yarns is the key factor affecting resistance–strain behaviors [28,29]. A knitted structure is composed of several yarn loops joined together. A single loop knitted structure contains three parts, termed the head, side limbs, and sinker loop, and different knitted structures have a different loop assembly and contact [30,31,32]. Hence, the length of the loop and the contact between each loop can be the main contributor affecting the resistance change and stable sensing ability of the conductive knitted fabric [25,33]. The change in resistance of the four different sample structures was examined to evaluate the sensitivity and stability under minimal tensile elongation.

**Figure 3:**
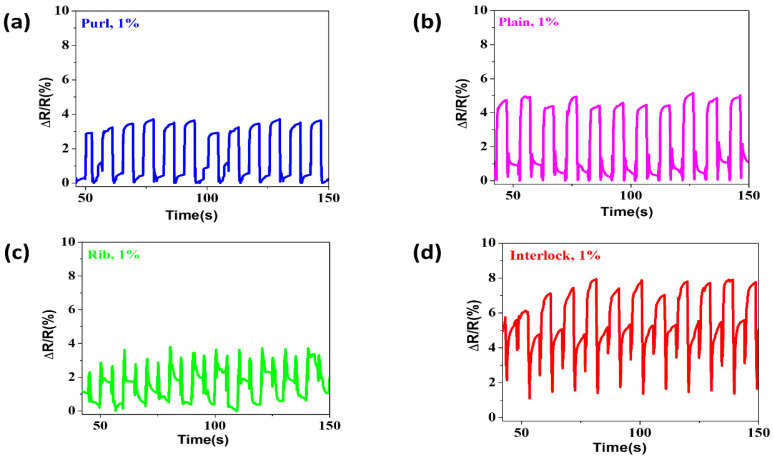
Strain response at 1% of elongation (**a**) purl, (**b**) plain, (**c**) rib, and (**d**) interlock structures.

During the experiment, two sides of the samples were covered by two copper electrode blocks to get precise and even changes of resistance during deformation of the fabric (Figure S4a). Conductive knitted fabrics mainly contain contact resistance, the resistance change of the samples was affected by the loop formation of the fabrics [34]. The yarn loop involves three main parts, and those create a contact point between each loop in a fabric (Figure S4b,c). While applying a tensile force to each sample fabric, contact pressure and friction force were generated between each loop, which existed at the contact point between the loops. Through a stretching and releasing action, a displacement of the contact point between each sample was created. The constancy in ∆R/R of the sample structures at 1% of tensile elongation is displayed in Figure 3. Theoretically, the change in resistance of the conductive knitted fabrics includes the resistance of the conductive fabric at a different time (R_t_) and the resistance at the initial time (R_0_) of the conductive fabric samples. Based on the following Equation (1) the relative resistance change (∆R/R (%)) of the samples was calculated, and all the sample structures were sensitive under low tensile force.

(1) ΔRR(%)=Rt−RoRo×100

Markedly different stabilities and uniformities were observed among each sample structure. Noticeably the different loop formations made the contact point greatly dissimilar. The purl structures can create constant movement and contact because of the lined connection of conductive yarns, yet for the other three structures, the assembly of conductive yarns might cause a broken contact because of the uneven distribution of yarns in the fabric surface. The purl structure revealed better stability and consistency in the change because of the even connection and distribution of the conductive yarn on the surface of the fabric compared to the other three knitted fabrics (Figure 3a).

The plain structure was the second best structure, this was perhaps because the backside of this structure is similar to the purl structure (Figure 3b). However, the rib and interlock structures had the most unstable change (Figure 3c,d).

The ∆R/R of the purl, plain, rib, and interlock was 1.62 ± 0.16, 2.16 ± 0.15, 1.06 ± 0.43, and 5.16 ± 1.06, respectively, which revealed that the resistance variation in interlock was the highest (Figure S5). The reason for this could be attributed to the structure’s loop formation and connection, with yarn overlaying and uneven spreading of the conductive yarns on the surface of the fabric. Hence, this affected the stability of ∆R/R and led to the formation of two change peak responses in 3% and 5% of plain, rib, and interlock with great unsteadiness (Figure S6).

**Figure 4:**
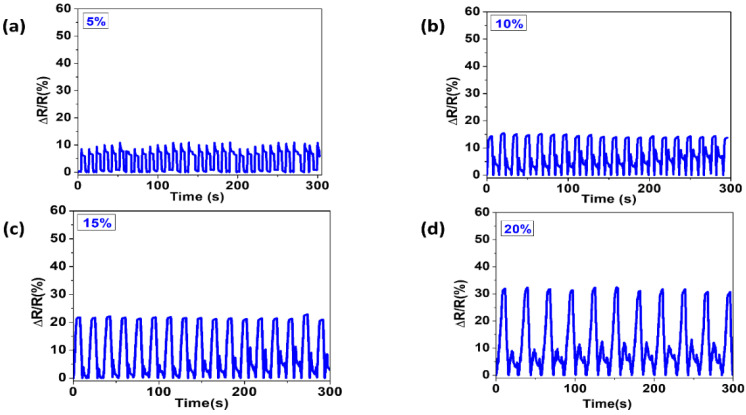
Change in resistance of purl structure under different strain percentage. (**a**) 5%, (**b**) 10%, (**c**) 15%, and (**d**) 20%.

The even distribution of the conductive yarn and the continual contact between loops of the purl structure led to a consistent change in resistance, starting at a low strain of 1%, to the higher 20% (Figure 4a–d).

### 3.3. Electro-Thermal Behavior and Temperature Distribution Test

**Figure 5:**
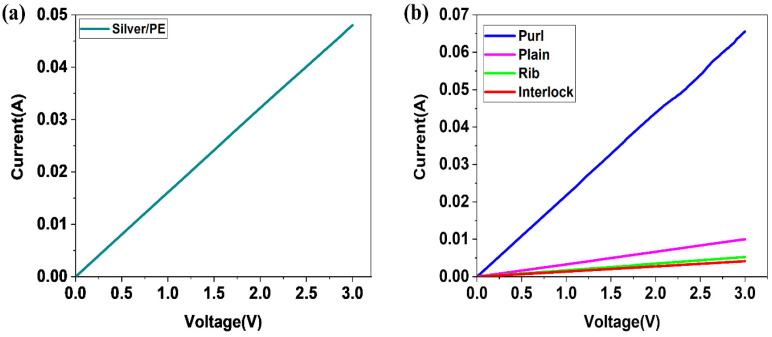
Current–voltage (*IV*) curves (**a**) silver/PE yarn, (**b**) different knitted structures.

In conductive materials, the main factors to be examined are the resistance, current, and voltage. Those factors can help clarify the different properties and behaviors of conductive materials. To determine the electro-thermal behavior of a material, it is vital to examine the current flow of the material. Current–voltage characteristic curves show a relationship between the current flowing through an electronic device and the applied voltage across its terminals. In a pure resistance, the relationship between voltage and current is linear at a constant temperature, such that the current is proportional to the potential difference [35]. The current–voltage IV curve for the silver twisted PE at room temperature showed a maximum current of 0.05 A under the voltage 0–3 V (Figure 5a).

Furthermore, the IV character of the different structures where examined, and the purl structure showed a high current with the increase of voltage (Figure 5b).

The voltage power used and the amount of heat produced are critical in medical textiles. Using low voltage power to produce a desirable heat is necessary for heating textiles. Heating textiles are required materials in medical applications such as therapy, yet it is important to understand the amount of heat that is suitable for human skin. Human skin is sensitive to heat depending on the amount of heat on the skin surface. Usually, the heat tolerated by human skin is between 40 and 44 °C, and higher temperatures can cause serious damage [36,37]. Nevertheless, the previously reported heating textiles were mainly focused on the production of high temperatures (>50 °C), and for this reason the voltage power utilized was >4 V. [13,38,39]. The drawback of applying high voltage power is that, not only can it produce a higher temperature than the needed amount, but it also could be destructive to human skin. This work scrutinized the electro-thermal properties and capabilities to produce an anticipated amount of heat, by utilizing voltage power from 1 V to 3 V in the knitted fabric.

**Figure 6:**
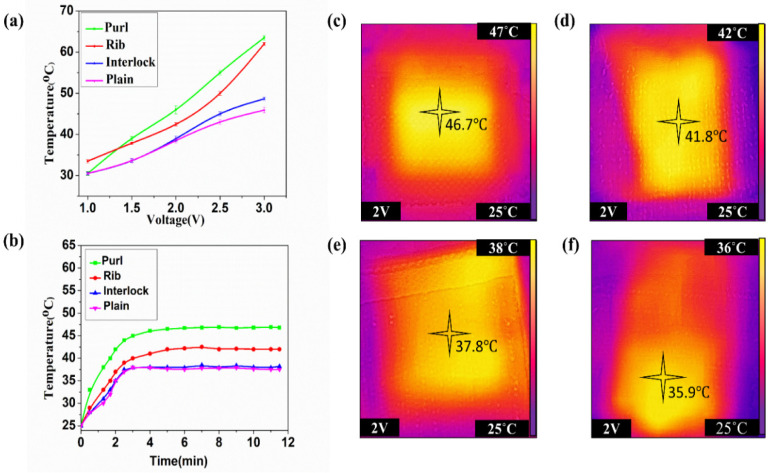
(**a**) Temperature change of different structures under different voltages, (**b**) at a constant voltage of 2V, thermal image of (**c**) purl, (**d**) rib, (**e**) interlock, and (**f**) plain.

The temperature distribution of each fabric structure that is integrated with conductive yarn was evaluated. Each experimental fabric was cut into a specimen with a size of 4 cm × 4 cm, and exposed to different voltage values from 1 V to 3 V with time, as shown in Figure 6. Once voltage was applied to the conductive knitted fabric, the entire specimens were heated, resulting from the effect of Joule heat. However, the distributions of heat were disparate amid each structure. The temperature was different for each sample depending on the structure and loop connection. Therefore, in the process of heating the samples, the purl structure was relatively stable compared to the other three structures. The temperature change of the purl structure was almost linear, as shown in Figure 6a. Moreover, according to the Joule heating effect, the lower the resistance of a material, the higher the current and power under a given voltage. Therefore, the purl structure, with a low resistance value can produce a higher current and power compared to the other three structures. The conductive loop yarns in the purl structure have an unbroken distribution and connection between each other, and this made the temperature change linear. Furthermore, in the heating garment, it is desirable if there is no fluctuation of the heating effect. The purl structure could reach 31 °C at 1 V and 64 °C at 3 V, which was the highest. Moreover, the rib was 33 °C at 1 V, 60 °C at 3 V, interlock was 30 °C at 1 V and 45 °C at 3 V, and the lowest was plain, being 30 °C at 1 V and 43 °C at 3 V. That is, under a low voltage power, purl could produce the needed temperature, and could not damage the skin. Figure 6b shows that all structures can produce reasonable temperatures under 2 V, yet smooth temperature distribution was vital. The purl structure was more regular compared to the other three structures, leading to a consistent heat distribution (Figure 6c). Once the samples were connected to the power sources, the surface of the entire fabric got warmer from one end to the other smoothly. Additionally, it had a uniform contact pressure and loop intersection, which led to a smooth distribution and change of temperature.

Even though the ratio of the yarns was the same (50/50) for both yarns, for all the structures, the structures formed diverse yarn expositions in the fabric. For the structures such as rib and interlock, the current flow in the structure was low. Hence both sides of the fabric got heated quickly and the rest of the fabric became warm after several minutes. As a result, the temperature change was affected, and it led to uneven temperature distribution, as shown in (Figure 6d,e).

Similarly, in the plain structure, because the back surface was mainly enclosed by the conductive yarn, it got warmer first, and after several minutes the front started to get warm (Figure 6f). The temperature change on both surfaces of the fabric was dissimilar, which effected the heat distribution. As the back of the fabric temperature reached maximal, the front side became only warm. Thus, the distribution became uneven.

**Figure 7:**
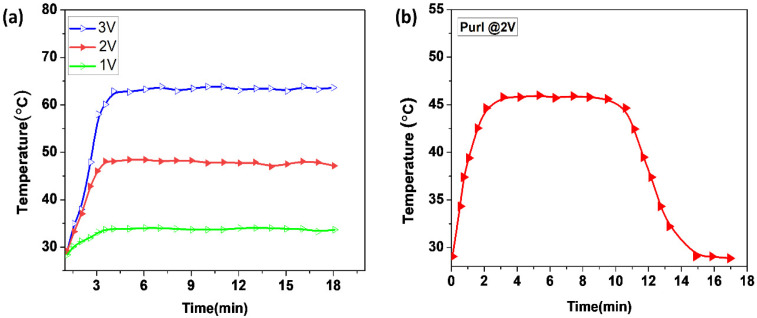
The temperature change of purl stitch (**a**) under 1 V to 3 V, (**b**) steadiness of temperature at 2 V and with time.

Hence, in the purl structure, the current flow between the loop structures was smoother and produced a suitable heat for wearable textiles. Although the temperature could reach as high as 65 °C at 3 V, the structure could provide the required temperature for RP patients at low voltage (Figure 7a). The rate of temperature change between each knit structure showed a variation result between different structures, and the change was disparate. All sample fabrics could be heated with time, yet the rate of change was dissimilar. As the structures showed, the conductive yarns were connected in a miscellaneous manner. The purl structure showed the highest rate of temperature change between 0 and 2 min, which was 45 °C distributed in the whole fabric, uniformly. Nevertheless, for the other three, the change rate from 0–2 min not only was lower than the purl but also the distribution was non-uniform. For example, in the plain structure, the change rate of the backside of the fabric was high compared to the front side. Similarly, the rib and interlock also had a non-uniform temperature change and distribution on the surface of the fabric between 0 to 2 min. The purl structure had a uniform and better temperature change rate, and a better temperature maintenance property.

This is a significant advantage in wearable smart textiles for the user and producer. The structure is auspicious for a wearable heating textile under low voltage power that can sustain the temperature for a longer time (Figure 7b).

### 3.4. Effect of Strain Force on the Temperature, and the Voltage Effect on the Resistance

**Figure 8:**
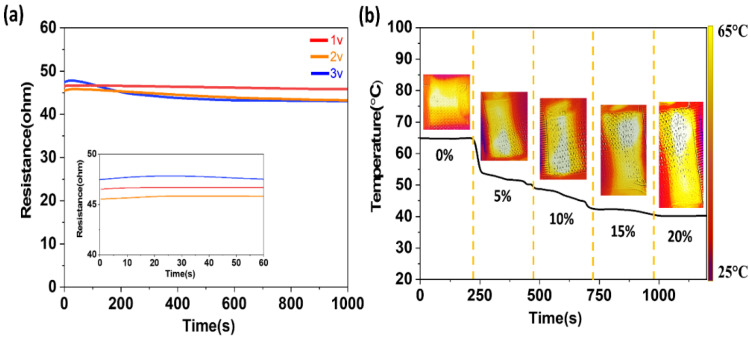
(**a**) Resistance of purl fabric under voltage values of 1 V, 2 V, and 3 V, (**b**) Temperature change under different strain forces (0–20%).

According to Joule’s law, the heat generated through applying power is one of the factors affecting resistance. Therefore, the reason for the change in the resistance measured by changing voltages may have been due to the temperature change. As the temperature increases, the resistance gradually decreased. Figure 8a shows that after a slight rise, during the very short initial period of the resistances in the inserted image, the resistance decreased during the heating process. The resistance of the purl fabric dropped when a different value of voltage was applied, and within the time it continuously and slowly decreased during the rest of the heating time. The higher the voltage applied, the smaller the resistance became. However, the amount of the resistance drop was not significant. Furthermore, the temperature versus strain of purl fabric was also investigated under strains from 0% to 20% at a constant voltage of 3 V. During the electro-heating change, under different strains the electric heating performance decreased slightly with a large strain. However, the temperature of the fabric was still 40 °C at 20% of strain (Figure 8b). The amount of heat generation was still suitable for an electro-thermal glove under a large strain.

**Figure 9:**
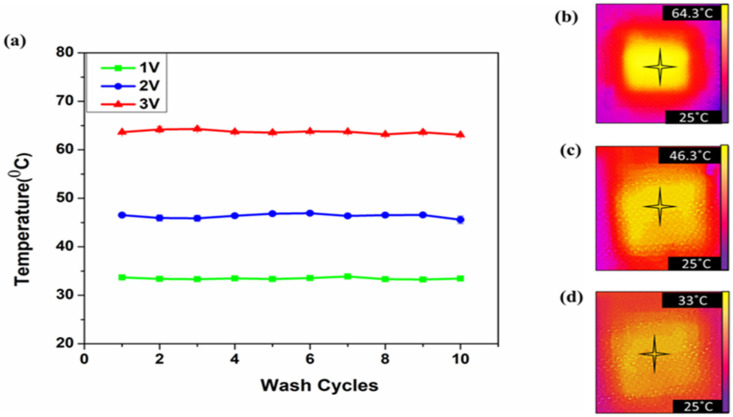
Temperature change (**a**) after 10 washing cycles using voltage values of 1 V, 2 V, and 3 V, (**b**–**d**) thermal image after washing.

In the meantime, it is important to study the washability and the effect of water on the electro-thermal behaviors of the fabric, since this material is used mostly in a moist and watery environment. The water or detergents used to wash, and the washing cycle can affect the performance of a conductive fabric [40]. Yet, the purl fabric was able to maintain an even temperature distribution after several washing cycles. Figure 9a–d illustrates the temperature change without significant change after a ten-wash cycle.

### 3.5. Design of the Electro-Thermal Glove with Sensor Function

Knitted fabric has a wide range of applications in the medical, sport, and other fields because of its properties [41]. Lately, there has been significant attention on developing knitted textiles that can be used to detect human motion or signal monitoring, but it is challenging to fabricant strain sensors that can give a uniform signal, are friendly to the environment, and conform to the human body [28,42,43]. The strain sensing performance of the purl fabric to human body motion was executed by attaching the fabric to a different part of the body. The conductive purl fabric was fabricated targeted to be used as a heating textile, as well as to provide sensor functions for different human body motions. The fabric texture was possibly compatible with human skin, and gave smooth detection signals in responding to the strain force. During elongation and release actions, the electrical resistance of the sensor mends as conductive materials recover to their original states or structures [44]. Knit based strain sensors have a great sensing capacity.

**Figure 10:**
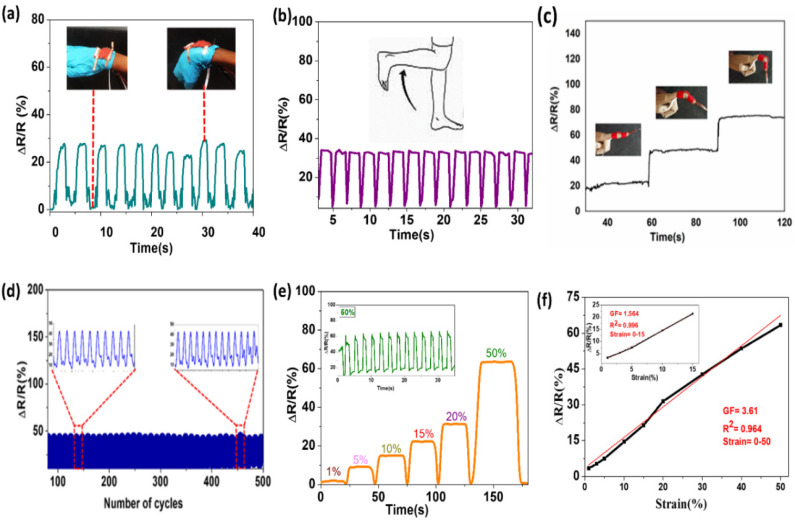
Applications of conductive purl fabric: (**a**) wrist bending, (**b**) knee bending, (**c**) finger bending at different degrees, (**d**) repeated cycle of strain force under 30%, (**e**) strain force at 1%, 5%, 10%, 15%, 20%, and 50%, and (**f**) gauge factor of purl fabric.

Sometimes it can be difficult to monitor complicated human body deformations because of multiple direction movements, and to precisely detect these using a strain sensor [44,45]. However, the purl structure revealed a stable response towards ∆R/R at several strain forces, to sense the motion of different parts of the human body. The fabric also was attached to some parts of the human body, such as the finger, wrist, and knee, to detect activities (Figure 10). As a result, this structure could be used to sense movements, from a slight movement of fingers, to a higher bending of the knee. It was able to uniformly respond to the bending of the wrist and the human knee (Figure 10a,b), and the movement of the finger at different bending degrees (Figure 10c). Therefore, the purl fabric presented a high potential for medical use, as well as for sportswear, during jogging to perceive signals, and as a glove to read finger and wrist movements. The fabric was also put under 30% of strain force for a repetitive 500 cycles, and it was able to preserve the signal detection consistency, without alteration (Figure 10d). Strain forces from 1% to 50% were applied to the purl structure, and the fabric was able to maintain the properties without alteration (Figure 10e). Furthermore, the gauge factor (GF), which is the most important index for the strain sensor, was calculated based on Equation (2), where ∆R/R is the change in resistance and ε is the strain. As a result, the strain ranges of 0−50, and GF showed a good fitting degree (Figure 10f). As the inserted figure in Figure 10f shows, the GF from 0–15% of strained showed an excellent fitting degree.

(2) GF=ΔRRΔLL=ΔRRε

**Figure 11:**
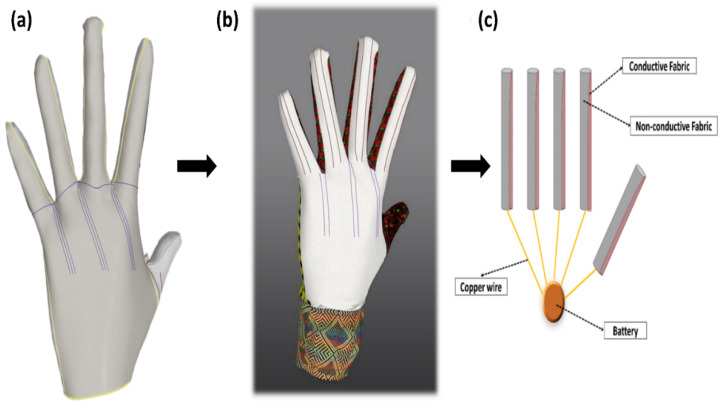
Thermal glove for RP patients: (**a**) wool glove interline, (**b**) glove design, and (**c**) glove assembly.

As was mentioned at the beginning of this work, the main symptoms of this disease are swelling and pain at the tip of the patient’s finger during cold weather and emotional variation [1]. This indicates that the rest of the finger is healthy. Therefore, it is preferable to insert the conductive fabric on one side of the tips of each finger, as shown in Figure 11. Likewise, to reduce the influence of the direct contact of the conductive fabric with the skin, it was important to insert a thin layer of wool fabric inside of the glove (Figure 11a).

This can give an additional heating function, while at the same time protecting the skin from undesirable and uncomfortable contact between the skin and fabric. Figure 11b,c demonstrate the final design of the electro-thermal glove, and the glove assembly, respectively. Therefore, after taking necessary consideration of the properties of the purl structure, it was decided to use it for the construction of the glove for RP patients. By using the marvelous designer computerized (M9) designing software the glove was designed considering the requirements of RP disease.

Normally, the finger becomes numb and inflexible during extreme cold and pain [46]. Thus, for RP patients while there is restricted blood and oxygen conveyance to the fingertips, it becomes stiff, numb, and painful to move the fingers. The glove is designed to be used as an electro-thermal glove to relax the patients’ blood vessels, and sense the motion on the finger after relaxation. Therefore, this glove can be a great help to read the movement of the fingertips after providing heat. Consequently, it is important to detect finger movements during and after the blood vessels are relaxed by the heat provided through the electrothermal glove.

The designed conductive fabric is joined with a non-conductive wool fabric in the fingertip, and the palm and wrist will be covered with pure wool yarn. The conductive silver/PE yarn was selected to reflect the heat and help prevent the loss of body heat, and the wool yarn provides warmth and comfort to the wearer. Moreover, the fabric was connected with a low voltage battery using copper wire. The purpose of this glove is to give warmth to the wearer and help the patient to relax the blood vessels and reduce pain. Furthermore, this glove is made to be used in any place and occasion for functional purposes and fashion wear, and it is affordable to all economic levels of user.

## 4. Conclusions

In summary, four different weft-knitted conducive fabrics were manufactured by collaborating wool yarn and silver/PE yarn using a computerized flat knitting machine. The conductive fabrics with different structure designs, termed plain, purl, interlock, and rib exhibited diverse results in heating performance. In addition, the sensor functionality of each fabric was explained. The purl structure showed better performance on uniform sensitivity to ∆R/R, strain force, temperature change, and distribution compared to the other three structures. The loop formation and conducive yarn distribution in both the front and backside of the purl structure was uniform, which led to a consistent change in resistance and temperature distribution. Thus, the purl structure was found to be an excellent contender to produce a heating glove for RP patients, and to detect the movement of several parts of the human body. Overall, this electro-thermal and sensor glove will have the potential to be used as a practical, fashionable, and comfortable textile that can be worn on any occasion.

## Data Availability

The data presented in this study are available on request from the corresponding author.

## Supplementary Materials

The following are available online at https://www.mdpi.com/1996-1944/14/2/377/s1, Table S1: Yarn specifications parameter, Table S2: Fabric structural, Figure S1: SEM image of (a) Wool yarn (b) PE yarn (c) Silver yarn (d) Twisted silver and PE yarn (e) silver yarn image, Figure S2: Tensile strength of wool and Silver/PE yarn, Figure S3: Image of fabric structures (a) purl, (b) plain, (c) interlock, (d) rib, Figure S4: (a) Machine setup of tensile force experiment, (b) knitted loop parts, (c) image of fabric stretching, Figure S5: The standard deviation of fabric resistance change, Figure S6: Strain response at 3% of elongation (a) plain, (b) rib, (c) interlock structure, and at 5% elongation (d) plain (e) rib and (f) interlock.

## References

[1] Fardoun M.M., Nassif J., Issa K., Baydoun E., Eid A.H. (2016). Raynaud’s Phenomenon: A Brief Review of the Underlying Mechanisms. Front. Pharmacol..

[2] Agbor V.N., Njim T., Aminde L.N. (2016). Difficulties in diagnosis and treatment of severe secondary Raynaud’s phenomenon in a Cameroonian woman: A case report. J. Med. Case Rep..

[3] Wigley F.M., Herrick A.L., Flavahan N.A. (2014). Raynaud’s Phenomenon: A Guide to Pathogenesis and Treatment.

[4] Temprano K.K. (2016). A Review of Raynaud’s disease. Mo. Med..

[5] Carlsson I., Cederlund R., Höglund P., Lundborg G., Rosén B. (2008). Hand injuries and cold sensitivity: Reliability and validity of cold sensitivity questionnaires. Disabil. Rehabil..

[6] Herrick A.L. (2017). Evidence-based management of Raynaud’s phenomenon. Ther. Adv. Musculoskelet. Dis..

[7] Lis-Święty A. (2019). Recent advances in the workup and management of Raynaud’s phenomenon. Pol. Arch. Intern. Med..

[8] Kim S.-W., Kim S.H., Kim C.S., Yi K., Kim J.-S., Cho B.J., Cha Y. (2020). Thermal display glove for interacting with virtual reality. Sci. Rep..

[9] Keef C.V., Kayser L.V., Tronboll S., Carpenter C.W., Root N.B., Finn M., O’Connor T.F., Abuhamdieh S.N., Davies D.M., Runser R. (2020). Virtual Texture Generated Using Elastomeric Conductive Block Copolymer in a Wireless Multimodal Haptic Glove. Adv. Intell. Syst..

[10] Wilson J., Wilson J. (2001). 10—Weft knitting, weft-knitted fabric and knitwear design. Handbook of Textile Design.

[11] Zhang X., Ma P. (2018). Application of Knitting Structure Textiles in Medical Areas. Autex Res. J..

[12] Cao X., Halder A., Tang Y., Hou C., Wang H., Duus J.Ø., Chi Q. (2018). Engineering two-dimensional layered nanomaterials for wearable biomedical sensors and power devices. Mater. Chem. Front..

[13] Zhang L., Baima M., Andrew T.L. (2017). Transforming Commercial Textiles and Threads into Sewable and Weavable Electric Heaters. ACS Appl. Mater. Interfaces.

[14] Husain M.D., Kennon R., Dias T. (2014). Design and fabrication of Temperature Sensing Fabric. J. Ind. Text..

[15] Åkerfeldt M., Strååt M., Walkenström P. (2012). Electrically conductive textile coating with a PEDOT-PSS dispersion and a polyurethane binder. Text. Res. J..

[16] Hao D., Xu B., Cai Z. (2018). Polypyrrole coated knitted fabric for robust wearable sensor and heater. J. Mater. Sci. Mater. Electron..

[17] Ma R., Lee J., Choi D., Moon H., Baik S. (2014). Knitted Fabrics Made from Highly Conductive Stretchable Fibers. Nano Lett..

[18] Kim H., Lee S., Kim H. (2019). Electrical Heating Performance of Electro-Conductive Para-aramid Knit Manufactured by Dip-Coating in a Graphene/Waterborne Polyurethane Composite. Sci. Rep..

[19] Hee A.C., Choudhury D., Nine M.J., Abu Osman N.A. (2014). Effects of surface coating on reducing friction and wear of orthopaedic implants. Sci. Technol. Adv. Mater..

[20] Yang Z., Pang Y., Han X.-L., Yang Y., Ling J., Jian M., Zhang Y., Yang Y., Ren T.-L. (2018). Graphene Textile Strain Sensor with Negative Resistance Variation for Human Motion Detection. ACS Nano.

[21] Liu S., Tong J., Yang C., Li L. (2017). Smart E-textile: Resistance properties of conductive knitted fabric–Single pique. Text. Res. J..

[22] Zhang Y., Long H. (2020). Resistive network model of the weft-knitted strain sensor with the plating stitch-Part 1: Resistive network model under static relaxation. J. Eng. Fibers Fabr..

[23] Liu S., Liu Y., Li L. (2019). The impact of different proportions of knitting elements on the resistive properties of conductive fabrics. Text. Res. J..

[24] Capineri L. (2014). Resistive Sensors with Smart Textiles for Wearable Technology: From Fabrication Processes to Integration with Electronics. Procedia Eng..

[25] Li L., Liu S., Ding F., Hua T., Au W.M., Wong K.-S. (2012). Electromechanical analysis of length-related resistance and contact resistance of conductive knitted fabrics. Text. Res. J..

[26] Ding J.T.F., Tao X., Au W.M., Li L. (2014). Temperature effect on the conductivity of knitted fabrics embedded with conducting yarns. Text. Res. J..

[27] Li L., Au W.M., Hua T., Wong K.S. (2011). Design of a conductive fabric network by the sheet resistance method. Text. Res. J..

[28] Seyedin S., Razal J.M., Innis P.C., Jeiranikhameneh A., Beirne S., Wallace G.G. (2015). Knitted Strain Sensor Textiles of Highly Conductive All-Polymeric Fibers. ACS Appl. Mater. Interfaces.

[29] Zhang H., Tao X., Yu T., Wang S. (2006). Conductive knitted fabric as large-strain gauge under high temperature. Sens. Actuators A Phys..

[30] Bueno M.-A., Camillieri B. (2019). Structure and mechanics of knitted fabrics. Structure and Mechanics of Textile Fibre Assemblies.

[31] Atalay O., Kennon W.R. (2014). Knitted strain sensors: Impact of design parameters on sensing properties. Sensors.

[32] Cooke B., Au K.F. (2011). 2—The physical properties of weft knitted structures. Advances in Knitting Technology.

[33] Zhang Y., Long H. (2020). Resistive network model of the weft-knitted strain sensor with the plating stitch-Part 2: Resistive network model during the elongation along course direction. J. Eng. Fibers Fabr..

[34] Xie J., Long H., Miao M. (2016). High sensitivity knitted fabric strain sensors. Smart Mater. Struct..

[35] Huzyak P., Ferguson J., Sharpensteen J., Xu L., Ananthakrishnan S.J., Rathnayake H. (2015). Fused arene-functionalized polyhedral oligomeric silsesquioxanes as thermoelectric materials. RSC Adv..

[36] Wienert V., Sick H., Mühlen J.Z. (1983). Local thermal stress tolerance of human skin. Anasth. Intensiv. Notf..

[37] Siekmann H. (1989). Determination of maximum temperatures that can be tolerated on contact with hot surfaces. Appl. Ergon..

[38] Huang J., Li Y., Xu Z., Li W., Xu B., Meng H., Liu X.-Y., Guo W. (2019). An integrated smart heating control system based on sandwich-structural textiles. Nanotechnology.

[39] Yang M., Pan J., Xu A., Luo L., Cheng D., Cai G., Wang J., Tang B., Wang X. (2018). Conductive Cotton Fabrics for Motion Sensing and Heating Applications. Polymer.

[40] Hardy D., Rahemtulla Z., Satharasinghe A., Shahidi A.M., Oliveira C., Anastasopoulos I., Nashed M.N., Kgatuke M., Komolafe A., Torah R. (2020). Wash Testing of Electronic Yarn. Materials.

[41] Yeoman M.S., Reddy D., Bowles H.C., Bezuidenhout D., Zilla P., Franz T. (2010). A constitutive model for the warp-weft coupled non-linear behavior of knitted biomedical textiles. Biomaterials.

[42] Wang Z., Chen J., Cong Y., Zhang H., Xu T., Nie L., Fu J. (2018). Ultrastretchable Strain Sensors and Arrays with High Sensitivity and Linearity Based on Super Tough Conductive Hydrogels. Chem. Mater..

[43] Zhong W., Liu C., Xiang C., Jin Y., Li M., Liu K., Liu Q., Wang Y., Sun G., Wang D. (2017). Continuously Producible Ultrasensitive Wearable Strain Sensor Assembled with Three-Dimensional Interpenetrating Ag Nanowires/Polyolefin Elastomer Nanofibrous Composite Yarn. ACS Appl. Mater. Interfaces.

[44] Yang K., Song G.-L., Zhang L., Li L.-W. Modelling the Electrical Property of 1×1 Rib Knitted Fabrics Made from Conductive Yarns. Proceedings of the 2009 Second International Conference on Information and Computing Science.

[45] Wang J., Lu C., Zhang K. (2020). Textile-Based Strain Sensor for Human Motion Detection. Energy Environ. Mater..

[46] Cheung S.S. (2015). Responses of the hands and feet to cold exposure. Temperature.

