# Supplementary material for: Design of Electro-Thermal Glove with Sensor Function for Raynaud’s Phenomenon Patients
Source: Materials (Basel). 2021 Jan 14;14(2):377. doi: 10.3390/ma14020377 (PMC7828797; doi:10.3390/ma14020377)
Supplement: Supplementary file 1 [file materials-14-00377-s001.pdf]

materials

Design of Electro-Thermal Glove with Sensor Function for Raynaud's Phenomenon Patients

Hewan Dawit [1,2], Qian Zhang [1,2], Yimeng Li [1,2], Syed Rashedul Islam [1], Jifu Mao [1,2,*] and Lu Wang [1,2,*]

1 Key Laboratory of Textile Science & Technology of Ministry of Education and College of Textiles, Donghua University, 2999 North Renmin Road, Songjiang District, Shanghai 201620, China; hewan27dawit@gmail.com (H.D.); zh901026@163.com (Q.Z.); yimeng_u@163.com (Y.L.); sri060791@gmail.com (S.R.I.)
2 Key Laboratory of Textile Industry for Biomedical Textile Materials and Technology, Donghua University, Shanghai 201620, China
* Correspondence: jifu.mao@dhu.edu.cn (J.M.); wanglu@dhu.edu.cn (L.W.)

Table S1. Yarn specifications parameter.

Material	Yarn thickness	Composition	Linear Resistance (ohm/cm)	Supplier
Silver twisted Yarn	250D/70D	PE(Polyester) twisted with Silver	1.96	Suzhou TEX Silver fiber technology CO, Ltd
Wool Yarn	$26^S/2$	100% Wool	Non-conductive	Hebei ASEAN Cashmere products CO, Ltd

Table S2. Fabric structural

Structure name	Number of stitches Crosswise/cm	Number of stitches longitudinally/cm	Loop length (mm)	Resistance(Ω/cm)	Thickness(mm)
Plain	8	11	3	48.8	1.3
Purl	7	7	3	45.5	2.0
Rib	5	8	6	47.9	2.2
Interlock	7	10	5	52.5	2.3

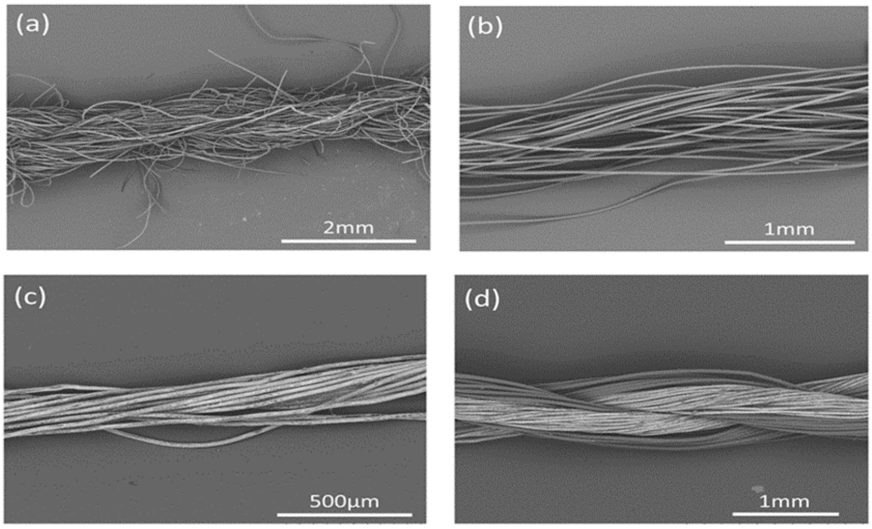

Figure S1. SEM image of (a) Wool yarn (b) PE yarn (c) Silver yarn (d) Twisted silver and PE yarn (e) silver yarn image.

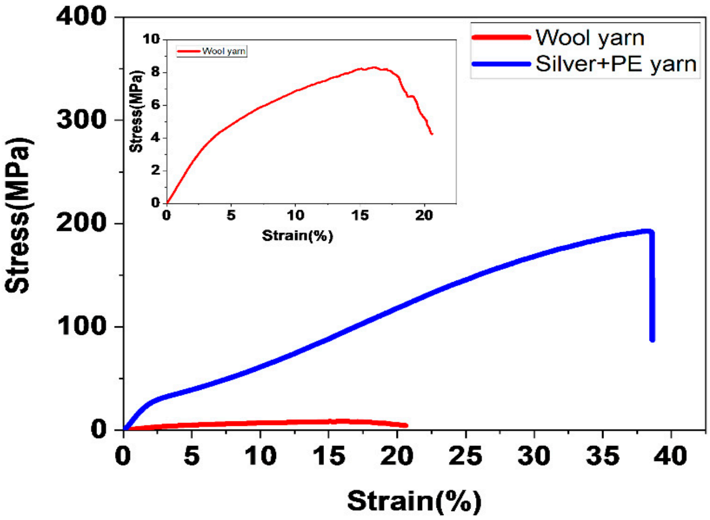

Figure S2. Tensile strength of wool and Silver/PE yarn

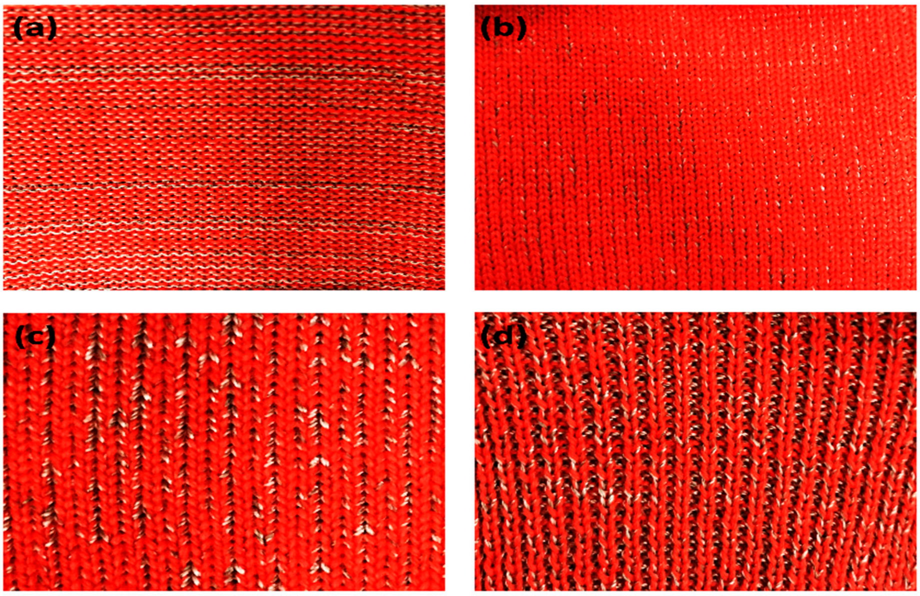

Figure S3. Image of fabric structures (a) purl, (b) plain, (c) interlock, (d) rib

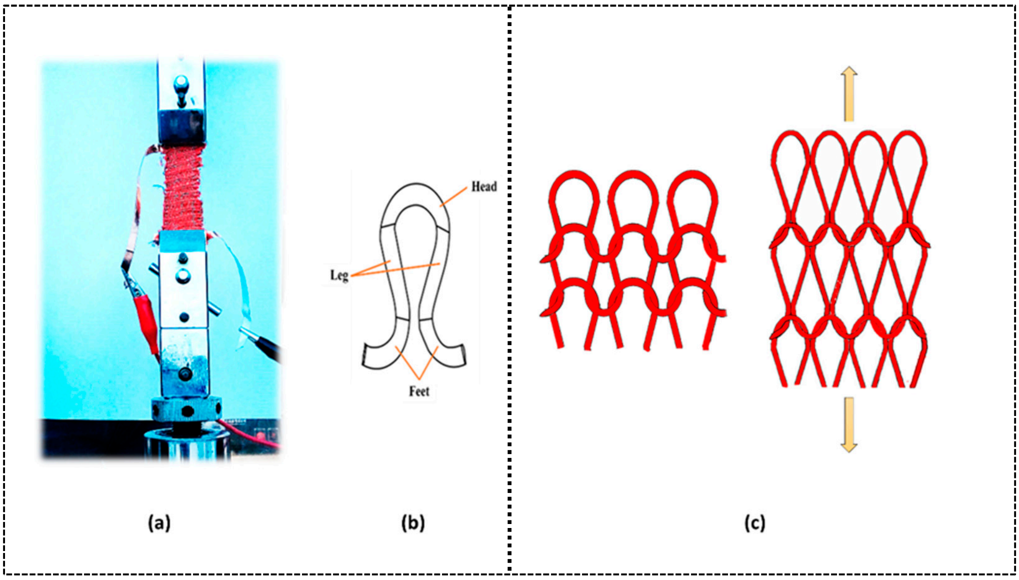

Figure S4. (a) Machine setup of tensile force experiment, (b) knitted loop parts, (c) image of fabric stretching

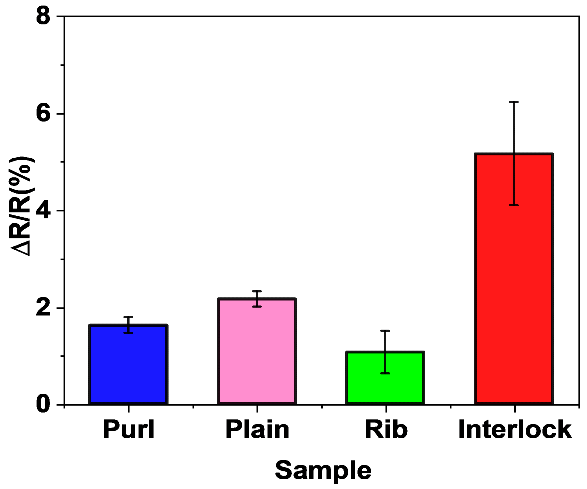

Figure S5. The standard deviation of fabric resistance change.

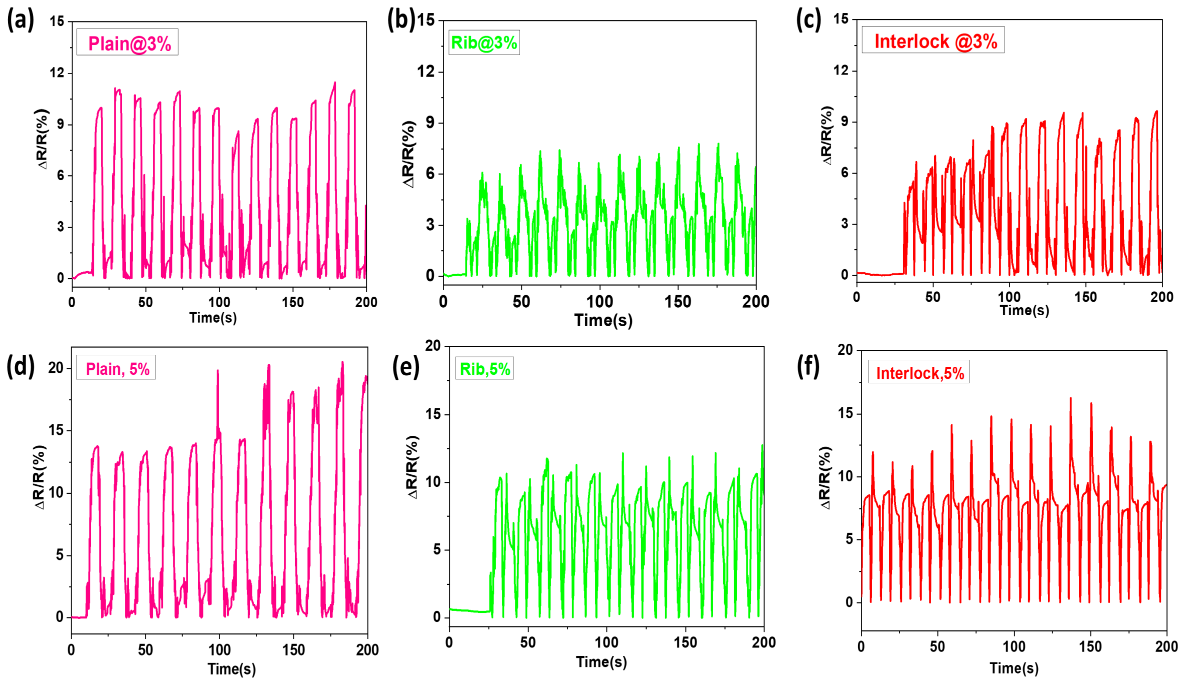

Figure S6. Strain response at 3% of elongation (a) plain, (b) rib, (c) interlock structure, and at 5% elongation (d) plain (e) rib and (f) interlock.
